# Parent‐Led Pain Management in Neonatal Care—Time to Move Forward

**DOI:** 10.1111/apa.70597

**Published:** 2026-05-15

**Authors:** A. Ullsten, F. Bacchini, M. Campbell‐Yeo, M. Eriksson, M. Lenells, E. Mezzalira, M. Mäki‐Asiala, E. Olsson, A.‐K. Palomaa, T. Pölkki, M. Rajala, A. Axelin, the PEARL Research Group

**Affiliations:** ^1^ Center for Clinical Research Region Värmland Karlstad Sweden; ^2^ Faculty of Medicine and Health, School of Health Sciences Örebro University Örebro Sweden; ^3^ Canadian Premature Babies Foundation Toronto Canada; ^4^ School of Nursing, Faculty of Health Dalhousie University Halifax Nova Scotia Canada; ^5^ MOM‐LINC Lab IWK Health Halifax Halifax Nova Scotia Canada; ^6^ Swedish Premature Infant Association Stockholm Sweden; ^7^ Department of Women's and Children's Health Karolinska Institute Stockholm Sweden; ^8^ Karolinska University Hospital Solna Sweden; ^9^ Department of Women's and Children's Health University of Padova Padova Italy; ^10^ Research Unit of Health Sciences and Technology University of Oulu Oulu Finland; ^11^ MRC Oulu Oulu University Hospital and University of Oulu Oulu Finland; ^12^ Department of Pediatrics, Faculty of Medicine and Health Örebro University Örebro Sweden; ^13^ Finnish Premature Infant Association Helsinki Finland; ^14^ Department of Nursing Science University of Turku Turku Finland

## 1 Introduction

As stated in the most widely ratified international human rights treaty in 1990, the Convention on the Rights of the Child (Articles 3, 4 and 5), infants have the right to be cared for by their parents (birthing person/caregiver(s)) and shall not be separated from their parents. The best interests of the child shall always be the primary consideration while also respecting the responsibilities, rights and duties of the parents. The devastating effects of parent–infant separation on infants' and children's emotional well‐being during routine hospital care were recognized and contested as early as the 1940s and 1950s [1, 2]. Although the child's access to comforting parents' presence has improved, there are still significant attitudes and beliefs among health care providers that continue to limit parental presence and involvement in neonatal care, with significant variation in practice globally [3].

Infants rely on their parents not only for survival and development, but also for comfort and protection. As recently as the 1980s, the mainstream view was that the infant brain was too immature to perceive or remember pain and, consequently, pain management was neither prioritized nor consistently integrated into standard care. Moreover, if pain care was offered, parental involvement was rarely emphasized, and procedures were often conducted in the absence of parents. Sadly, despite high‐quality evidence demonstrating the effectiveness of parent‐led pain care, global data indicate that many infants—both healthy and ill—continue to undergo painful needle‐related procedures without parental presence or involvement in procedural support [4]. Urgent action is needed to protect infants' rights to parental presence and the inclusion of parents as *partners* in neonatal care management [3].

As the saying goes, ‘It takes a village to raise a child’, but where is the support from the ‘village’ during the many painful procedures newborns endure as part of neonatal care? In reality, although neonatal care is often viewed as a shared responsibility, support for parent involvement during painful procedures remains limited and acceptance and implementation of parent‐led pain management remain suboptimal. A recent global survey of parent‐led pain management in neonatal care found that, although two‐thirds (67%) of the responding units (*n* = 303) had local neonatal pain guidelines, only 40% of those recommended parent‐led interventions or parental involvement in pain assessment [5].

Grounded within our ongoing multinational research project POP (Parent‐led pain management to Optimize neonatal Pain care, www.pearl.direct/home/pop), funded by Nyckelfonden Research Foundation at Örebro University Hospital, the main aim of this paper is to advocate for a call for action to improve infant pain management through the inclusion of parents during all stages of management including shared decision‐making regarding assessment, plan, provision and evaluation. Current evidence related to the efficacy, acceptability and feasibility of parent‐led neonatal pain care, as well as barriers and facilitators related to implementation, will be discussed.

## 2 Parents' Perspective on Neonatal Pain

The POP Study was co‐designed by parents, researchers and clinicians in a common effort to improve neonatal pain management. The neonatal period is defined as care provided to any infant less than 44 weeks postnatal age and includes those healthy, sick or preterm. Parent partners within POP bring extensive first‐hand experience with the evolution of evidence‐based neonatal pain management guidelines. Moreover, as both parents and advocates, they have observed and have lived experience that these guidelines frequently fail to translate into consistent bedside clinical practice.

‘I felt so helpless because I felt I could not do anything to help my child when she was in the neonatal intensive care unit (NICU) for pain relief. I trusted doctors and nurses in everything. When I got the opportunity to have my child in kangaroo care I felt I could really do something for her well‐being’. Mari, mother (Finland)

‘A routine vaccination during the NICU stay became a source of grief long after the event. Despite agreeing with the nurses that we would be there and wanted to be there, the vaccination was done out of convenience without us parents. It was devastating not being able to be there to comfort my child knowing that she was alone and knowing how negative pain is for brain development’. Mikaela, mother (Sweden)

‘Not being involved in Gabriel's pain management in the NICU still affects me today. He was poked every day—one day, 13 times—and I was not ‘allowed’ to be present to hold him or comfort him. I was once told that babies do not feel pain. We now know that preterm infants not only feel pain, but that repeated, unmanaged procedural pain can influence brain development and long‐term outcomes. As a parent, learning this after the fact is devastating. Had I understood the evidence then, I would have advocated to be present for every procedure, to provide skin‐to‐skin care, and to be an active partner in his pain management plan’. Fabiana, mother (Canada)

## 3 Latest Evidence on Parent‐Led Pain Management

Preventing pain by reducing the number of performed procedures and avoiding routine blood sampling are effective strategies for lowering infants' cumulative pain exposure [6]. Transferring agency and responsibility to the parents to assess pain and deliver pain management is a key component of pain prevention.

A substantial and growing body of evidence supports that parent‐led pain management—implemented in collaboration with healthcare providers and supported by shared decision‐making, including parental involvement in pain assessment—significantly reduces procedural pain associated with repeated early‐life interventions [3, 6, 7]. Importantly, most parents report that they are willing to take on this responsibility if supported by clinicians [8]. Culturally sensitive biopsychosocial strategies like the parent‐led interventions may reduce the need for additional pharmacological pain treatments, particularly those associated with a higher risk. These approaches can be safely extended to home and community settings, further reinforcing parental confidence and caregiving roles [6, 7].

Parent‐led pain interventions, such as skin‐to‐skin contact, breastfeeding and live singing, are humane, culturally informed and equitable, while also being effective, feasible and cost‐efficient. These interventions can be tailored to the needs of both the infant and parent. Moreover, parent‐led pain management aligns with family‐integrated care, a progressive philosophy and model of neonatal care that positions parents as primary caregivers and integral members of the care team [9].

Collaborative planning of procedures, along with shared roles in pain assessment and management, has been shown to reduce stress and pain in both infants and parents, while enhancing comfort and well‐being. Evidence further demonstrates that interprofessional collaboration that includes parents as active participants improves procedural pain outcomes in infants [10].

In conclusion, parent‐led interventions are effective, easy to use with little to no risk and implementable into everyday clinical practice in both low‐ and high‐resource settings [11, 12, 13].

## 4 Successes and Shortcomings in the Uptake of Parent‐Led Pain Management

In the Nordic countries, family‐friendly societal policies and strong commitments to gender equity in caregiving reinforce each infant's right to parental presence during hospitalization. Across Sweden, Finland, Norway, Denmark and Iceland, awareness and implementation of family‐centred care are well established and continue to advance, with parents mostly welcomed and routinely included in their infant's daily care.

Most Nordic neonatal units have been purpose‐built or redesigned to support continuous parental presence, featuring single‐family rooms, zero‐separation policies, bedside rounds and accommodations for siblings. Publicly funded healthcare and comprehensive social security systems are important factors in the sustainability of family‐centred care, where hospitalized children have a legal right to have at least one parent present during hospitalization.

In Nordic NICUs, parent‐led pain management, combining skin‐to‐skin contact, breastfeeding and parental live singing, is on the clinical agenda supported by innovative collaborative research initiatives involving parent‐staff‐researcher partnership [11, 12]. Evidence from these initiatives demonstrates that individualized preparation and education enable meaningful parent participation and promote infant–parent closeness during procedures, resulting in effective pain and stress reduction [11, 12]. Partnership and collaboration are central to successful implementation.

In Canada, in their pain and prevention clinical practice statement, the Canadian Paediatric Society has provided the first global paediatric declaration that provides clear prioritization of parent‐led strategies based on empiric evidence [6, 7]. It affirms that all health care providers caring for infants (healthy, small and/or sick) have a responsibility to provide effective pain management and emphasizes that parent‐led interventions—among the most effective approaches—should be prioritized.

Despite strong evidence supporting parent‐led pain management in NICUs, its integration into clinical practice remains limited due to slow implementation of evidence‐based practice [4]. One key factor underlying both successes and shortcomings is the quality of parent–staff communication, which determines how well parents are informed about their important role, receive guidance and feel empowered to seize the opportunity [14, 15]. It is a growing process for parents to take their role in the interprofessional collaboration around the infant's pain management [14]. However, healthcare professionals often make assumptions about parental readiness without directly assessing it. Parents frequently report that information about pain management is insufficient, poorly timed or not aligned with their emotional readiness, while clinicians may act as gatekeepers due to concerns about parental anxiety or uncertainty about preparedness [15]. These barriers limit parents' active involvement, even in settings that endorse family‐integrated care. Strengthening communication through staff education, consistent and timely information‐sharing, and trust‐based, respectful interactions is essential to enhance parent‐led pain care [15].

Advancing effective and equitable implementation of parent‐led pain management requires diligent efforts to enhance communication, clarify parental roles and embed shared decision‐making within clinical culture.

## 5 Time to Move Forward

Facilitating parent‐led neonatal pain management is the central aim of the international research and dissemination initiative POP (www.pearl.direct/home/pop), built by parents, researchers and clinicians. This work challenges traditional hierarchies in neonatal care by addressing power imbalances and enabling those most affected—particularly parents—to shape decisions and solutions. By shifting the role of researchers from experts to collaborators, POP promotes shared authority in defining priorities and advancing care.

‘Parents are not passive observers in the NICU. We are protective regulators for our children, and our involvement in pain mitigation is both emotionally essential and scientifically supported. However, thirteen years later, on a recent hospital re‐admission with my son, I found myself navigating the same painful gap between what should happen and what actually happens. When pain prevention measures are missed, it is us parents who carry the emotional weight and, too often, the responsibility to speak up. Guidelines matter, but only when they are lived in practice’. Fabiana, mother (Canada)

In the best interest of the vulnerable infants and their parents, there is an urgent need for action to uphold infants' and parents' rights to optimal pain care, now. The POP research group calls for global implementation to ensure that every infant, regardless of birthplace, has access to parent‐led pain management during common painful procedures.

## Author Contributions

**A. Ullsten:** conceptualization, writing – original draft, writing – review and editing, validation. **F. Bacchini:** writing – review and editing, validation. **M. Campbell‐Yeo:** writing – original draft, writing – review and editing, validation. **M. Eriksson:** writing – original draft, writing – review and editing, validation. **M. Lenells:** writing – review and editing, validation. **E. Mezzalira:** writing – review and editing, validation. **M. Mäki‐Asiala:** writing – review and editing, validation. **E. Olsson:** writing – review and editing, validation. **A.‐K. Palomaa:** writing – review and editing, validation. **T. Pölkki:** writing – review and editing, validation. **M. Rajala:** writing – review and editing, validation. **A. Axelin:** conceptualization, writing – original draft, writing – review and editing, validation.

## Funding

This study was funded by Nyckelfonden Research Foundation at Örebro University Hospital.

## Conflicts of Interest

The authors declare no conflicts of interest.

## Data Availability

Data sharing not applicable to this article as no datasets were generated or analysed during the current study.
